# Objective Video Quality Assessment Based on Machine Learning for Underwater Scientific Applications

**DOI:** 10.3390/s17040664

**Published:** 2017-03-23

**Authors:** José-Miguel Moreno-Roldán, Miguel-Ángel Luque-Nieto, Javier Poncela, Pablo Otero

**Affiliations:** Department of Communication Engineering, University of Málaga, 29071 Málaga, Spain; luquen@uma.es (M.-Á.L.-N.); jponcela@uma.es (J.P.); pablo.otero@uma.es (P.O.)

**Keywords:** objective video quality assessment, machine learning, MOS, VQA, QoE

## Abstract

Video services are meant to be a fundamental tool in the development of oceanic research. The current technology for underwater networks (UWNs) imposes strong constraints in the transmission capacity since only a severely limited bitrate is available. However, previous studies have shown that the quality of experience (QoE) is enough for ocean scientists to consider the service useful, although the perceived quality can change significantly for small ranges of variation of video parameters. In this context, objective video quality assessment (VQA) methods become essential in network planning and real time quality adaptation fields. This paper presents two specialized models for objective VQA, designed to match the special requirements of UWNs. The models are built upon machine learning techniques and trained with actual user data gathered from subjective tests. Our performance analysis shows how both of them can successfully estimate quality as a mean opinion score (MOS) value and, for the second model, even compute a distribution function for user scores.

## 1. Introduction

Video quality assessment (VQA) is an important aspect of multimedia services in any communication network. The understanding of how users perceive quality enables us to study the feasibility of potential services and adapt the network available resources to satisfy the user requirements. Subjective quality assessment is usually regarded as the most reliable technique to evaluate a multimedia service since it is based on data gathered from opinion tests with human viewers. However, these tests usually involve a considerable amount of resources (mainly time and people) and are considered expensive. Objective quality assessment methods provide an alternative approach built upon mathematical estimators of the quality. They usually require a set of subjective scores to build the estimation model, but then they can compute quality without further human intervention. Objective methods also yield the same quality estimation every time a certain video sample is given as input. This is not true for subjective methods due to the inherent nature of human judgements.

Information gathered by underwater networks (UWNs) is currently focused on the measurement of physical and chemical magnitudes, but multimedia information is a crucial instrument in the study of the oceans, and video services could become a key application. The deployment of wireless sensor networks capable of video capturing and transmission would be a significant technological improvement allowing for continuous monitoring of underwater environments or cooperative exploration with autonomous underwater vehicles (AUVs). A video service with enough quality could lead to decisions about the re-planning of an AUV path even if instant remote control were not possible due to transmission delays. However, current underwater wireless networks experience limited capabilities. Electromagnetic propagation cannot be used for communication ranges beyond centimeters due to the high absorption of the water. Acoustic communication is used instead, but the state of the art acoustic modems reach a peak data rate of 62.5 kbps with a 300 m operating range [1]. Bitrates available in the application layer are even smaller and the quality is seriously burdened by this constraint. In [2] the authors have already shown that video services are feasible, even with the high constraints of the current technology, but within a small range of the considered input variables the quality jumps from the bottom to the top of the quality scale. This agrees with conclusions presented in [3] where the authors state that models for objective quality assessment achieve a better performance when they are tuned for a combination of human and system factors. In this context, a set of solid VQA tools is a key aspect for any potential video service provided in UWNs. These tools could be used as estimators of the achievable quality of a service during the network design stage, but also as real-time estimators of the quality and, thus, a way to optimize such quality with respect to changing conditions. However, the limitations of the environment should be taken into account and the scarcity of resources calls for an optimized VQA technique. If quality monitoring is carried out on the receiver side, the reduced network bitrate heavily burdens the amount of extra information which can be transmitted for quality measuring purposes. The nodes should also be designed for energy efficient performance since batteries cannot be easily replaced; therefore, intensive processing tasks should be avoided if we want our nodes to compute the quality estimation and perform real-time adaptation.

The International Telecommunications Union (ITU) classifies objective VQA methods according to the input information and the primary application of their models [4]. Media signal models are used for quality benchmarking and make use of the signals to predict QoE. Parametric packet-layer models and bitstream-layer models are both utilized for in-service nonintrusive monitoring; the first class only needs packet headers for quality estimation, while the second class also necessitates the payload information. Parametric planning models are employed for network planning or terminal/application design. These methods require quality design parameters and no actual signal is needed for the prediction. The last ITU class considers hybrid models that take as input any combination of the previous ones and are commonly used for in-service nonintrusive monitoring.

A different simpler classification can often be found in the literature [5,6,7,8]. This categorization focuses on the inputs used to yield the quality estimation. Full reference (FR) methods require the original video signal as well as the received version and compute the quality estimation by comparing both signals. Reduced reference (RR) techniques only require some portion of the original information (some extracted features) to perform the comparison with the received version. No reference (NR) VQA methods calculate the estimation without any knowledge of the transmitted video. NR methods can be pixel-based if they need to decode the video signal, or bitstream-based if they can compute the quality estimation without fully decoding the video signal (e.g., using the information in the packet headers).

FR methods seem unsuitable for UWNs since the original unimpaired signal cannot be monitored due to the location of the network nodes. Even a deferred acquisition of this data is unfeasible in the short or middle term since the recovery of a node after it is deployed is expensive and should be delayed until its energy is depleted. RR methods can be used if the amount of data to be sent along with the video signal is small in comparison to the available bitrate. Considered video bitrates for UWSN are below 20 kbps [2], therefore only a few bytes per second would be available for VQA purposes. NR methods seem also suitable in this environment since no extra information is transmitted.

The term “machine learning” originates in the field of artificial intelligence theory. Now, these techniques are widely used in other fields and the term can be generalized to any statistical tool that can be used to build a model for a process without explicitly programming the outcomes. In this sense, any algorithm that is able to learn from data, starting with the simplest linear regression, is a machine learning technique. Machine learning algorithms are usually grouped in the broad categories of classification and regression. In the first case, the algorithm will learn to assign a class to the input data. A typical example of classification in the field of image processing is learning to detect a pathology from a medical image, from a set of samples where the presence or absence of the pathology is known. In the second case the algorithm can learn to estimate a value for a certain input, given a set of known input values and their corresponding output values. A typical example of a regression model in the field of image processing is learning to estimate the future size of a tumor from a medical image, given a set of images of the development of similar tumors. Since we have available a dataset with subjective quality scores for underwater video [2], we can leverage the power of the machine learning approach to build regression models for objective quality estimation.

The remaining of this paper is organized as follows: Section 2 presents a survey on the state of the art of RR and NR methods for VQA. Section 3 describes the experimental dataset which serves as input data for the analyses in this study. Section 4 examines the suitability of the ITU standardized method for parametric NR VQA. The definition of the proposed models and the corresponding performance analyses are presented in Section 5 (NR model) and 6 (RR model). Finally, Section 7 contains the conclusions drawn from this research work.

## 2. Related Work

Most of the existing bibliography on objective VQA focuses on mean opinion score (MOS) estimation. The MOS statistic stems from subjective quality tests. In these studies, the users issue a score for every video sample in a categorical quality scale. A five-class scale (bad, poor, fair, good and excellent) is often employed and classes are mapped to numerical values (1–5) for easier processing. The MOS is the average of these values across all the users for each sample. Objective VQA methods estimate this value because it is an intuitive and easy to use quality metric.

There are several pixel-based and bitstream-based good performing NR methods available [7,8,9,10]. Nonetheless, all of them involve a considerable amount of image or feature extraction processing. This processing load can be considered reasonable for typical computing capabilities, even for inexpensive equipment. However, energy saving is a priority in UWSNs and intensive processing tasks should be avoided, as mentioned above.

Some parametric network planning methods can also be found in the literature [11,12,13,14,15,16,17,18,19,20]. These techniques are lighter in processing since they only require the evaluation of a function to compute the quality estimation. A performance comparison between all of them was conducted in [21], concluding that the best results for encoding impairments are obtained with [20] and the best results for transmission impairments are achieved with the ITU standard G.1070 [22]. However, the procedure proposed in [20] is not strictly a parametric method since the video content is introduced in the model with the average sum of absolute differences (SAD) per pixel and therefore actual video signals should be used to compute the quality estimation.

Machine learning techniques have also been applied successfully to the problem of VQA. The work in [23] describes a RR method using a convolutional neural network which is usually regarded as one of the machine learning procedures with a higher computational cost [24]; Another study [25] proposes a NR support vector machine (SVM) regression but, again, a moderate amount of processing is required to extract the eighteen different features necessary for the estimation. Similar problems can be found in [8] where the number of features increases up to fifty-four. A decision tree is trained in [26] to develop a NR bitstream-based method but the work focuses on a subjective dataset with a very high coding bitrate to resolution ratio, which greatly differs from our environment.

MOS has already been found an insufficient metric unable to provide information about user diversity; however, a number of investigations have tried to overcome this limitation. A very interesting approach to QoE research is offered in [27], where a model with additional statistics is provided; it shows how the MOS hides relevant information. Nevertheless, QoE is addressed generally and video services are only included as a use case. Moreover, the authors state that these particular services do not fully fit the binomial distribution of scores proposed in the paper. Another noteworthy effort to overcome the MOS limitations has been done in [28]. The authors use machine learning techniques to build a prediction model for the proposed metrics: the degree of general acceptability and the degree of pleasant acceptability. Yet, it is based on a non-standard subjective data experiment which requires a complex procedure.

None of the works mentioned in this section take into account the scarcity of resources we have described for UWNs nor do they consider the differences in user perception which have been observed in scientific applications [2]. In this paper, we present two machine learning models for quality estimation designed for underwater video. The first of them is a NR parametric planning model based on surface fitting regression techniques. This model is able to provide a computationally fast and lightweight estimation of quality with only two service parameters (bitrate and framerate). The second model goes beyond MOS and computes estimations of full score distributions from the same service parameters and two video content features and thus it is a RR hybrid model. Ordinal logistic regression serves as a machine learning foundation algorithm for this model which also produces quality predictions with lightweight processing.

## 3. Subjective Dataset

The experimental data used for model fitting and machine learning in the present paper are part of the results obtained in a previous subjective test that has been already published in [2]. This test was performed according to the ITU standards for subjective quality assessment of video services ITU-R BT.500 [29] and ITU-T P.910 [30]. In the test, all the human subjects (viewers) were ocean scientists. They were presented a collection of videos with different features and were asked to score them in a standard five-rank quality scale: bad, poor, fair, good and excellent. All video features in our dataset were selected according to the constrained transmission capabilities already mentioned for underwater networks (see Section 1). The bitrate is the main limiting factor, so the remaining settings have to agree with that restriction. The results were statistically processed and MOS values were computed for every set of features. Analysis of variance (ANOVA) tests were used to compute *p*-values and assess the statistical significance of the results. The conclusions in [2] show that bitrate, frame rate as well as content variation are the input variables in the test that have a higher impact on the quality score and will be thus selected as features for machine learning algorithms. The conclusions in [2] also validate the feasibility of the selected video settings with fairly high quality scores and link this quality to the usefulness regarded by ocean scientists in the test.

The metrics for content variation are the spatial and temporal perceptual information (SI and TI) as defined in (1) and (2) [30]. They are only applied to the luminance plane of the images. The Sobel operator in (1) is a convolutional kernel operator used for edge detection [31]. The std_space_ operator computes standard deviation of luminance values within a single pixel matrix. The max_time_ operator selects the maximum value of the argument (spatial standard deviation for a pixel matrix in both cases) over the set of all processed video frames in the clip. The features of the video clips selected for this paper are shown in Table 1. Other video features kept constant for all the clips were H.264 compression format, RGB (24 bits) color and QVGA (320 × 240) resolution. Clips are grouped for comparison purposes in two blocks according to their content variation: a high variation content (HVC) block and a low variation content (LVC) block.

Additionally, an alternative, reduced, low variation content block (rLVC) will be considered. This block contains every point in the LVC block except rows with ID 06 and 07 (see Table 1). Another conclusion drawn in [2] relates quality to usefulness for the specific application of scientific underwater video. The MOS values for clips 06 and 07 break the trend of the whole dataset and it is possible that the particular content of these clips shifted the opinion of the viewers since some starfish can be seen in clip 06, while clip 07 contains plain seafloor with a few scattered small cavities. Although further subjective tests should be conducted to assess this hypothesis, it is useful to consider the rLVC block as a tool to avoid overfitting:

(1) $$SI=\max_{time}\left\{std_{space}\left[Sobel\left(F_{n}\right)\right]\right\}$$

(2) $$TI=\max_{time}\left\{std_{space}\left[M_{n}\left(i,j\right)\right]\right\},\;\mathrm{with}\;M_{n}\left(i,j\right)=F_{n}\left(i,j\right)-M_{n-1}\left(i,j\right)$$

## 4. G.1070 Model Suitability Study

The VQA model proposed in [11] is, to the best of our knowledge, the only parametric model standardized by ITU as part of the ITU-T G.1070 recommendation: “Opinion model for video-telephony applications” [22]. Although it was designed for this specific application some authors consider it a general reference for parametric models [21]. The model computes a MOS value from a group of equations which take as input parameters the bitrate (Br), the frame rate (Fr) and the packet-loss rate. The model coefficients must be selected according to some other service variables: the compression codec, the video resolution and the physical display size. The recommendation provides five “provisional” coefficient sets in Appendix I (not an integral part of the recommendation), which can be used under some restrictions regarding bitrate and packet loss. Due to these restrictions, only coefficients in sets #1 and #2 could be used in underwater video, but the MOS values predicted by the model do not correlate with our subjective data as shown in [2]. Therefore, the first step in the search for a parametric model for underwater VQA is calculating a new set of coefficients for the G.1070 model.

According to the available information in our experimental data, the G.1070 model can be simplified as shown in Equations (3)–(6), where MOS is the quality prediction in the usual 1–5 MOS scale, *Ofr* is the optimal frame rate for a given bitrate, *I_Ofr_* is the maximum video quality for a given bitrate and *D_Fr_* is the degree of robustness due to the frame rate. This model does not take into account content variation and therefore SI, TI information must be discarded:

(3) $$MOS=1+I_{Ofr}\exp\left(\frac{{\left[\ln\left(Fr\right)-\ln\left(Ofr\right)\right]}^{2}}{2D_{Fr}^{2}}\right)$$

(4) $$Ofr=\;v_{1}+v_{2}B,\;1\leq Ofr\leq 3$$

(5) $$I_{Ofr}=\;v_{3}-\frac{v_{3}}{1+{\left(\frac{Br}{v_{4}}\right)}^{v_{5}}}\;,\;1\leq I_{Ofr}\leq 4\;$$

(6) $$D_{Fr}=\;v_{6}+v_{7}Br,\;0<D_{Fr}$$

Annex A in the recommendation specifies the methodology for deriving the coefficients from a subjective quality dataset. The procedure is based on successive least square approximations (LSA). The first step obtains, for each bitrate, estimations of intermediate parameters *Ofr*, *I_Ofr_* and *D_Fr_*, based on frame rate values. However, the LSA for our subjective data cannot be solved in the real domain as shown in Table 2. For the high variation content, the imaginary part of the intermediate parameters could be considered negligible since it is nine orders of magnitude smaller than the real part and thus the coefficients can be calculated with another LSA approximation. Table 3 contains these results along with the goodness of fit (GOF) standard measures: the sum of squares due to error (SSE), the R square (R^2^) and the root mean squared error (RMSE). All of them indicate a very poor fit quality with a negative R^2^ showing that even a simple linear regression (plane) would be more appropriate for the data. The poor performance of this model could be attributed to the fact that it was designed for a very specific application (video telephony) which greatly differs from underwater video services in several important aspects such as video content and features, purpose of the video service and user expectancies. These differences can considerably change user perception of quality.

## 5. NR Parametric Model (Surface Fitting Non-Linear Regression)

### 5.1. Deriving the Model

**Figure 1:**
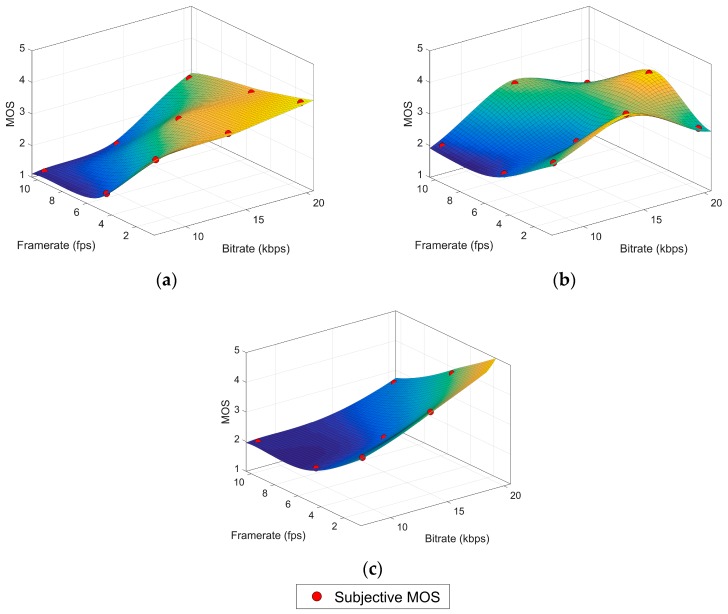
Thin plate spline surfaces. (**a**) HVC block, (**b**) LVC block, (**c**) rLVC block.

In the previous section we have shown that the ITU reference model does not seem suitable for the experimental data. As an approximation to an appropriate model, we have used the thin plate spline interpolation method [32] to find a surface for each of the content variation subsets. The thin plate spline is defined as the unique minimizer of the energy function defined in (7) for the two dimensional case. This method provides a perfect fit (R^2^ = 1) for the given control points. The minimization constraint produces a smooth surface (minimally “bended”) which matches the assumption of no great variations in quality values between the studied input variables values. The surface can be defined as in (8), a weighted sum of the radial basis function in (9) where *x^(i)^* are the control points, *K* is the number of points and *a_i_*, *w_i_* are the optimization parameters. In this case, the control points are the samples from our subjective dataset, considering the bitrate as our first dimension or feature (*x*_1_) and the framerate as the second feature (*x*_2_). The resulting MOS for a given sample is *y^(i)^*. This interpolation technique produces a representative surface but not the practical model we aim for, since the complexity of the resulting equation makes it difficult to interpret the coefficients. Figure 1 shows three surface plots of thin plate splines fitting the subjective dataset. Figure 1a is obtained from points in the high variation content (HVC) block as control points, while points in the low variation content (LVC) and reduced low variation content (rLVC) blocks are for Figure 1b,c, respectively. The shapes of the HVC and rLVC surfaces are very similar. Even the LVC surface could be regarded as reasonably similar, except for the bending forced by the anomalies already mentioned in Section 3. Our model proposal in the Section 5.2 is motivated by the resemblance between this geometrical profile and a sigmoid function.

(7) $$E_{tps}=\overset{K}{\underset{i=1}{\sum }}{\left|f\left(x^{\left(i\right)}\right)-y^{\left(i\right)}\right|}^{2}+\lambda \iint \left[{\left(\frac{\partial f^{2}}{\partial x_{1}^{2}}\right)}^{2}+2{\left(\frac{\partial f^{2}}{\partial x_{1}\partial x_{2}}\right)}^{2}+{\left(\frac{\partial f^{2}}{\partial x_{2}^{2}}\right)}^{2}\right]dx_{1}dx_{2},$$

where

(8) $$f\left(x_{1},x_{2}\right)=a_{0}+a_{1}x_{1}+a_{2}x_{2}\overset{K}{\underset{i=1}{\sum }}w_{i}\varphi \left(\left|\left(x_{1},x_{2}\right)-x^{\left(i\right)}\right|\right)$$

and

(9) $$\varphi \left(r\right)=r^{2}ln\left(r\right).$$

### 5.2. Model Equations and Discussion

Our first proposal is a regression model based on the generalized logistic function (10) [33]. The coefficients in the logistic function are relatively easy to relate to the function behavior and thus an interpretation of their values can be extracted. Our model extends the generalized logistic function for two dimensions and includes a linear function with a nonzero y-intercept term for the exponential in the denominator to improve the fitting performance. We propose two different variations of the model according to different optimization objectives:

1. Generalization—Non-linear regression model (NLR.G).
  Equation (11) achieves a more consistent behavior of the model outside the range of the subjective dataset. The asymptotes of the surface are set to the limits of the quality scoring scale (1–5).
2. Accuracy—Non-linear regression model (NLR.A).
  Equation (12) achieves a better fitting for the points in the subjective dataset (higher R^2^):
  (10) f(x)=L+U−L(A+Be−Cx)1v ,
  (11) f(x)=1+4(A)1v(A+Be−(c0+c1x1+c2x2))1v ,
  (12) f(x)=L+K(A+Be−(c0+c1x1+c2x2))1v .

**Figure 2:**
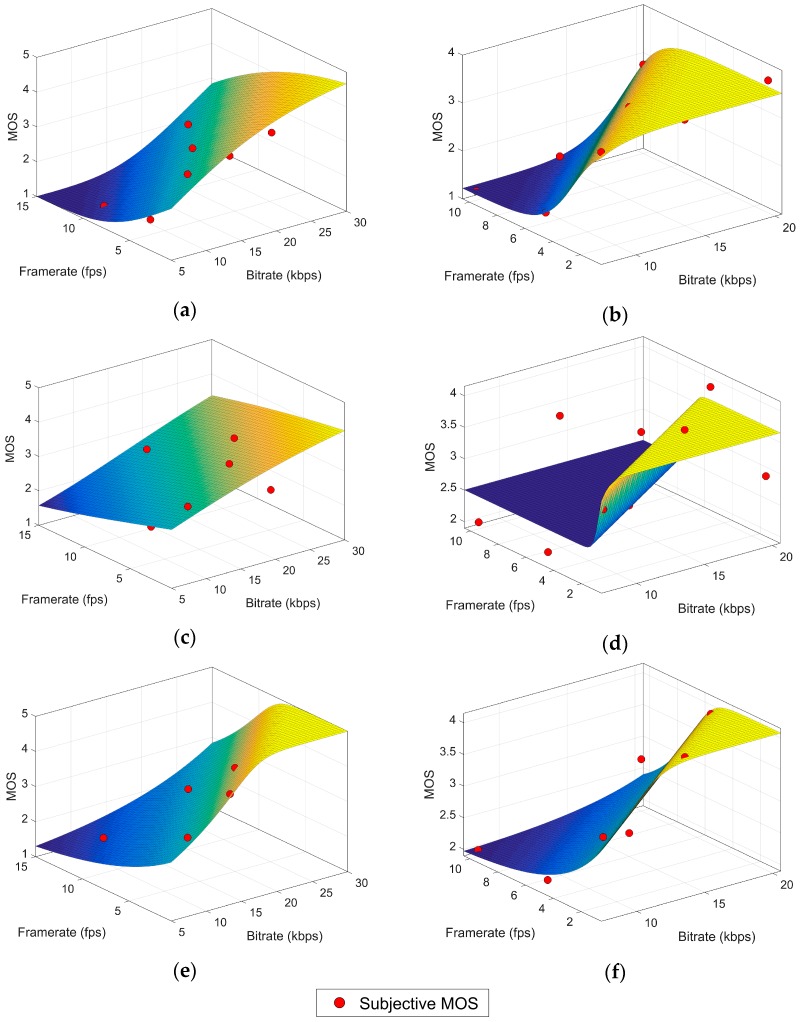
NR model surfaces. (**a**) NLR.G–HVC, (**b**) NLR.A–HVC, (**c**) NLR.G–LVC, (**d**) NLR.A–LVC, (**e**) NLR.G–rLVC, (**f**) NLR.A–rLVC. Note that the bitrate axis in (**a**,**c**,**e**) has been extended to show the generalization behavior.

Parameters *L*, *U*, *K*, *A*, *B*, *c_i_*, υ are optimized with the non-linear least squares method applied to our subjective dataset. The coefficients computed for every block can be found in Table 4 for the NLR.G model and in Table 5 for the NLR.A model. The corresponding goodness-of-fit statistics (SSE, R^2^, RMSE) are shown in Table 6 and Table 7. These values are used as a performance metric of the model. Figure 2 contains plots for each model surface: NLR.G surfaces are in the left column while the right column shows the NLR.A surfaces.

The model fit for high variation content video is very good for the NLR.G model (R^2^ ≈ 0.88) and excellent for the NLR.A model (R^2^ ≈ 0.98). The performance of the model is poor (R^2^ ≤ 0.6) for the low variation content videos because the model cannot fit the non-sigmoid shape of the cloud of points. However, the performance dramatically rises to the levels of high variation content for the reduced low variation content dataset, with an excellent performance of both NLR.G (R^2^ ≈ 0.91) and NLR.A (R^2^ ≈ 0.94). The RMSE value is also considerably low for both blocks (RMSE ≤ 0.5), taking into account that it is not being averaged over the total number of samples but over the difference between the number of samples and the number of parameters in the model.

Beyond goodness-of-fit considerations, NLR models offer an easily computable approach to objective quality assessment. An underwater node could obtain an estimation of the MOS in a fast and energy-efficient way since it only requires the video coding bitrate and framerate as input variables and no further calculation is needed.

## 6. RR Hybrid Model (Ordinal Logistic Regression)

In spite of the advantages of the NLR model, for some applications it could be regarded as too simplistic. Firstly, video content is only considered in a coarse way, as two big blocks of high and low variation content. Secondly, relevant data is lost when computing the MOS because the information about the distribution of scores is discarded when they are transformed into a single averaged value.

The ordinal logistic regression (OLR) [34,35] is a classification method for multiclass problems with a natural order among the response categories. Thus, it is perfectly suitable for the quality assessment experiment in which users issue scores within an ordered categorical scale (bad, poor, fair, good and excellent). In OLR a sample or observation *x* is a group of values of the input variables associated to a distribution of scores for the outcome variable. All the video features described in Section 3 will be used as inputs of the model. Therefore, each observation is a four-component vector including as features the bitrate (*x*_1_), the framerate (*x*_2_), the SI (*x*_3_) and the TI (*x*_4_). We call *π_i_(x)* the probability of the observation x to be in the *i*-th category. For k categories of the outcome variable, the method computes the *k−1* logarithms of the odd ratios or logits, i.e., the logarithms of the probability of being in a given category or any category below (*γ_j_*) divided by the probability of being in any superior category.

The model is based on the proportional odds assumption, which states that these logits can be represented by a linear model with a different intercept term *θ_j_* for every logit but the same coefficients *β* for all the predictors (13). The *π_i_(x)* probabilities are obtained from the model as in (14). An estimator for the MOS is proposed in (15). Even though our target was departing from the MOS simplistic approach to QoE assessment, the MOS estimator can still be useful for comparing with other models:

(13) $$log\left(\frac{\gamma_{j}\left(x\right)}{1-\gamma_{j}\left(x\right)}\right)=\theta_{j}+\beta^{T}x,\;where\;\gamma_{j}\left(x\right)=\sum_{i=1}^{j}\pi_{i}\left(x\right)$$

(14) $$\pi_{i}\left(x\right)=\gamma_{j}\left(x\right)-\gamma_{j-1}\left(x\right)=\frac{\exp\left(\theta_{j}+\beta^{T}x\right)}{1+\exp\left(\theta_{j}+\beta^{T}x\right)}-\frac{\exp\left(\theta_{j-1}+\beta^{T}x\right)}{1+\exp\left(\theta_{j-1}+\beta^{T}x\right)},\;\mathrm{with}\;\gamma_{5}=1,\;\gamma_{0}=0\;\forall x$$

(15) $$MOS_{OLR}=\overset{5}{\underset{i=1}{\sum }}i\times \pi_{i}\left(x\right)$$

The IBM SPSS Statistics [36] software has been used for the model fitting through maximum-likelihood estimation and for the analysis of results. Since it has been already shown that there are some interactions between the model inputs (i.e., the video features; see Section 3) [2], we have followed an iterative procedure to build our model. This procedure creates a model with as many interaction terms as possible. It, then, discards the non-significant interactions based on their *p*-value. A high *p*-value means the interaction is non-significant and it can be discarded; otherwise, the interaction term is retained. The procedure can be described with the following two-step loop:

For *i* = *num_features* to *i* = 1

1. Compute a model including every possible interaction except the ones that have been discarded in a previous iteration.
2. Check the *p*-value of the coefficient for every interaction term of *i-th* order (or main effect if *i* = 1). If *p* > 0.05, the interaction is considered non-significant and thus removed from subsequent iterations.

After this iterative procedure, the remaining main effects and interactions as well as the computed coefficients are shown in Table 8 along with the intercept term for each category. Table 9 collects the result of two χ^2^ tests. The “model fitting test” is a Likelihood Ratio χ^2^ test between a model with only the intercept term and the final model. The *p*-value for our model is significant (*p* = 0.005) and indicates that the final model fits the dataset better than a model with constant odds based on the marginal probabilities of each outcome category. The “parallel lines test” is an analogy between the final model and a multinomial model where no natural ordering is considered between categories and therefore different *β* coefficients are obtained for every logit estimator. The *p*-value is non-significant (*p* = 1.00) and thus there is no evidence to reject the assumption of proportional odds. Several R^2^ values are provided in Table 10.

**Figure 3:**
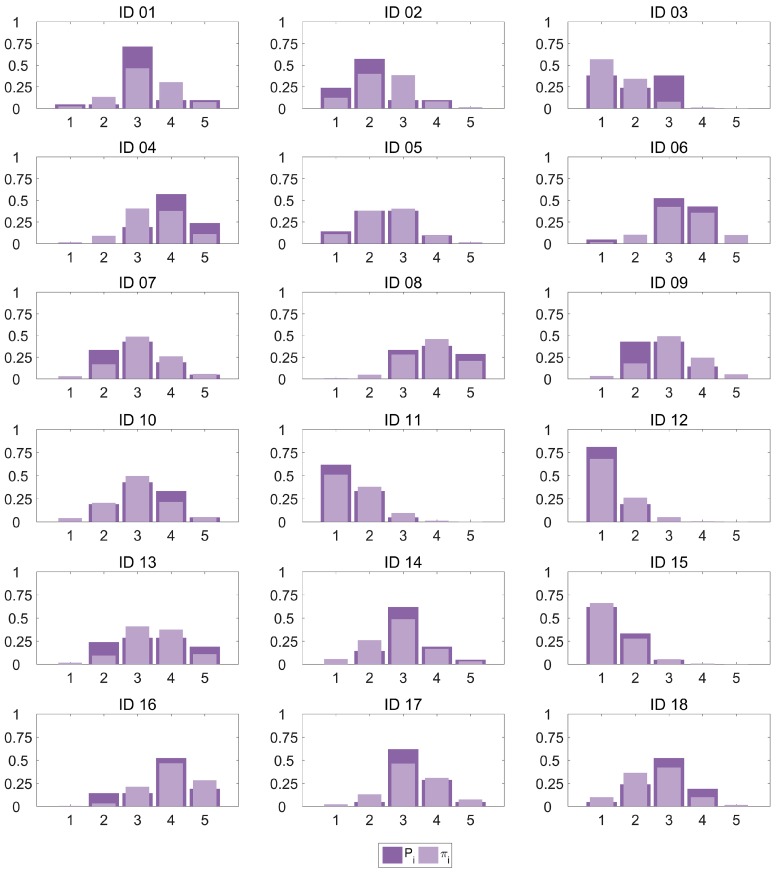
Proportions of scores from subjective data and estimated probabilities from OLR model.

We have computed an R^2^ value for the MOS_OLR_ estimator and the subjective MOS values in the dataset, resulting in a 90% of the variance explained by our estimator. This result is similar to the R^2^ obtained with the NR models. Pseudo-R^2^ values are also included in our results as Cox and Snell [37], Nagelkerke [38] and McFadden [39]. These pseudo-R^2^ values, as discussed in [40], cannot be interpreted like a classic R^2^ in a least squares regression since they do not provide a comparison between the predicted values and those in the dataset, but between the fitted model and the only-intercept model described above. However, they serve to compare different models.To provide a graphical approach to the goodness-of-fit, Figure 3 plots the category probability distribution *P_i_* for every observation in our dataset as estimated by the OLR model against the proportions of scores computed from the subjective data *π_i_*. It can be observed how the model provides a very good fit for most cases, with an excellent performance for some of the observations (IDs 05 and 15) and only a small amount of higher errors (category 3 in IDs 01 and 03, and category 2 in ID 09). In particular, 71.1% of the *π_i_* estimations show a deviation smaller than 0.1.

We could also consider the mode of the distribution (the value that occurs most frequently) of scores to be the best categorical guess for the quality. In this case, if we select the categories with max(*π_i_*(x)) and max(*P_i_*(x)) as the classification decision, the accuracy of the classification method is 83.3%.

## 7. Conclusions

Underwater video services greatly support oceanic research. Subjective studies show that useful services can be offered even with the important bitrate limitations imposed by the current technology. Objective quality assessment tools are essential in highly constrained environments, both in the network planning and service provisioning stages. They provide a method to identify the configuration parameters which make the difference between a useless service and a valuable one.

However, not every quality estimation method is suitable for this particular problem, due to the peculiarities of underwater communications: nodes are difficult to reach once deployed, the limited bandwidth does not allow for an extra communication channel for measuring purposes, and energy saving restrictions prevent the use of intensive processing tasks.

This paper exposes the unsuitability of the standardized ITU parametric method for underwater video quality estimation and presents two alternative models based on machine learning algorithms. These models are able to successfully accommodate the specific perception of quality revealed by subjective tests while taking into account the aforementioned special conditions. The first model is a parametric no reference estimation method and, therefore, only the evaluation of the model equation is required to predict MOS values. It shows a very good fit to the subjective data (R^2^ ≈ 0.9) and can be used for network planning applications but also to obtain a fast, lightweight processing estimation of the quality for real-time adaptation. The second model is a reduced reference method with a similar performance in terms of MOS prediction (R^2^ ≈ 0.9) but it further explores the concept of quality estimation. This technique, built upon ordinal logistic regression, is capable of predicting the distribution of user scores and thus provides a full characterization of quality beyond the simplistic common MOS statistic. This approach has not been previously applied to video quality assessment and delivers a more reliable way to assess user satisfaction and quality of experience. Future work is still required to determine the reliability of our models with other experimental datasets built with underwater video and subjective quality scores. However, there are currently no other datasets with these features publicly available to perform this comparison. Further research effort is also required to increase the number of underwater video quality databases.

## Figures and Tables

**Table 1:** Video features for model fitting and machine learning algorithms.

Block	ID	B_r_ (kbps)	F_r_ (fps)	SI	TI
LVC	01	8	1	23.95	4.46
	02	8	5	23.87	4.19
	03	8	10	24.35	4.38
	04	14	1	30.35	4.58
	05	14	5	27.66	4.16
	06	14	10	29.35	6.24
	07	20	1	41.46	7.18
	08	20	5	36.87	9.20
	09	20	10	39.23	7.13
HVC	10	8	1	67.13	15.42
	11	8	5	75.43	13.96
	12	8	10	57.69	13.46
	13	14	1	71.11	15.92
	14	14	5	66.52	13.92
	15	14	10	76.33	18.05
	16	20	1	71.11	15.92
	17	20	5	60.21	11.20
	18	20	10	53.95	10.15

**Table 2:** Intermediate parameter estimation for deriving coefficients of the G.1070 model.

Br (kbps)		8	14	20
**LVC**	**Ofr**	1.013 × 10^−7^	–30.7385 − 7.813 × 10^−8^ i	2.969 − 1.138 × 10^−13^ i
	**I_Ofr_**	31.826	2.219 − 0.07 i	3.336 − 1.206 × 10^−13^ i
	**D_Fr_**	6.906	14.01 + 2.155 i	1.05 − 8.857 × 10^−14^ i
**HVC**	**Ofr**	1.878	1.101	0.682
	**I_Ofr_**	4.955	2.204	0.577
	**D_Fr_**	2.43 + 1.066 × 10^−9^ i	1.688 − 5.507 × 10^−9^ i	1.811 + 6.434 × 10^−9^ i

**Table 3:** HVC coefficients for the G.1070 model and GOF statistics.

***v*_1_**	***v*_2_**	***v*_3_**	***v*_4_**	***v*_5_**	***v*_6_**	***v*_7_**
2.445	0.0459	1.946	7.935	32.431	–0.294	0.094
**SSE**		**R2**			**RMSE ^1^**	
36.9130		−0.0561			2.5906	

^1^ RMSE averaged over the difference between the number of samples and the number of parameters in the model.

**Table 4:** Coefficients for the NLR.G model.

Block	*A*	*c*_0_	*c*_1_	*c*_2_	*ν*
HVC	6.994	5.569	0.0977	–0.1512	3.623 × 10^−4^
LVC	487.1	–1.008	0.05259	−0.05686	5.195 × 10^−3^
rLVC	23.33	–15.31	0.7495	–1.224	10.37

**Table 5:** Coefficients for the NLR.A model.

**Block**	***L***	***K***	***A***	***B***
HVC	1.291	3.518	1.539	2.411
LVC	2.505	7.83	3.864	11.11
rLVC	1.933	2.264	1.362	4.158
**Block**	***c*_0_**	***c*_1_**	***c*_2_**	***ν***
HVC	−1.952	0.6349	−0.9421	1.013
LVC	−16.62	3.128	−6.671	0.7034
rLVC	−9.609	1.063	−1.906	5.672

**Table 6:** GOF statistics for the NLR.G model.

Block	SSE	R2	RMSE ^1^
HVC	0.959	0.8809	0.4896
LVC	2.687	0.3945	0.9186
rLVC	0.3916	0.9084	0.4425

^1^ RMSE averaged over the difference between the number of samples and the number of parameters in the model.

**Table 7:** GOF statistics for the NLR.A model.

Block	SSE	R2	RMSE ^1^
HVC	0.116	0.9856	0.34
LVC	1.936	0.5637	1.391
rLVC	0.2609	0.939	–

^1^ RMSE averaged over the difference between the number of samples and the number of parameters in the model.

**Table 8:** Coefficients for the OLR model.

**Category/Logit**	**Coefficient**	**Value**
$\frac{\mathrm{bad}}{\mathrm{poor}\;\mathrm{or}\;\mathrm{better}}$	***θ*_1_**	6.839
$\frac{\mathrm{poor}\;\mathrm{or}\;\mathrm{worse}}{\mathrm{fair}\;\mathrm{or}\;\mathrm{better}}$	***θ*_2_**	8.891
$\frac{\mathrm{fair}\;\mathrm{or}\;\mathrm{worse}}{\mathrm{good}\;\mathrm{or}\;\mathrm{better}}$	***θ*_3_**	11.066
$\frac{\mathrm{good}\;\mathrm{or}\;\mathrm{worse}}{\mathrm{excellent}}$	***θ*_4_**	13.097
**Effect/Interaction**		
Framerate	***β*_1_**	0.333
SI	***β*_2_**	−0.871
TI	***β*_3_**	0.607
Bitrate*Framerate	***β*_4_**	−0.083
Bitrate*SI	***β*_5_**	0.024
Framerate*SI	***β*_6_**	0.090
Framerate*TI	***β*_7_**	−0.318
SI*TI	***β*_8_**	0.037
Bitrate*SI*TI	***β*_9_**	−0.002

**Table 9:** Chi-Squared tests for the OLR model.

Test		−2 Log Likelihood	*χ*^2^	df *	p
Model fitting	Intercept only	485.514	–	–	–
	Final **	235.726	249.788	9	<0.005
Parallel lines	Null hypotesis **	235.726	–	–	–
	General	232.135	3.591	27	1.000

* Degrees of freedom. ** Fitted OLR model.

**Table 10:** Pseudo-R^2^ and R^2^ statistics for the OLR model.

p-R^2^–C&S *	p-R^2^–N **	p-R^2^–M ***	R^2^–MOS_OLR_
0.484	0.509	0.220	0.90

* Cox and Snell, ** Nagelkerke, *** McFadden.
